# The Molecular and Cellular Mechanisms of Axon Guidance in Mossy Fiber Sprouting

**DOI:** 10.3389/fneur.2018.00382

**Published:** 2018-05-29

**Authors:** Ryuta Koyama, Yuji Ikegaya

**Affiliations:** Laboratory of Chemical Pharmacology, Graduate School of Pharmaceutical Sciences, The University of Tokyo, Tokyo, Japan

**Keywords:** hippocampal mossy fiber, axon guidance, epilepsy, BDNF, netrin, dentate gyrus

## Abstract

The question of whether mossy fiber sprouting is epileptogenic has not been resolved; both sprouting-induced recurrent excitatory and inhibitory circuit hypotheses have been experimentally (but not fully) supported. Therefore, whether mossy fiber sprouting is a potential therapeutic target for epilepsy remains under debate. Moreover, the axon guidance mechanisms of mossy fiber sprouting have attracted the interest of neuroscientists. Sprouting of mossy fibers exhibits several uncommon axonal growth features in the basically non-plastic adult brain. For example, robust branching of axonal collaterals arises from pre-existing primary mossy fiber axons. Understanding the branching mechanisms in adulthood may contribute to axonal regeneration therapies in neuroregenerative medicine in which robust axonal re-growth is essential. Additionally, because granule cells are produced throughout life in the neurogenic dentate gyrus, it is interesting to examine whether the mossy fibers of newly generated granule cells follow the pre-existing trajectories of sprouted mossy fibers in the epileptic brain. Understanding these axon guidance mechanisms may contribute to neuron transplantation therapies, for which the incorporation of transplanted neurons into pre-existing neural circuits is essential. Thus, clarifying the axon guidance mechanisms of mossy fiber sprouting could lead to an understanding of central nervous system (CNS) network reorganization and plasticity. Here, we review the molecular and cellular mechanisms of axon guidance in mossy fiber sprouting by discussing mainly *in vitro* studies.

## Introduction

Dentate granule cells project unmyelinated axons, i.e., mossy fibers, through the dentate hilus to the CA3 region of the hippocampus (1). These mossy fibers first converge in the hilus and give rise to several collaterals that extensively branch out and contact the dendrites of hilar neurons, including the mossy cells and pyramidal basket cells. After exiting the hilus, the mossy fibers project through CA3 toward the border between CA3 and CA2. In CA3, the largest fraction of the mossy fibers project through a narrowly bundled area above the CA3 pyramidal cell layer called the stratum lucidum, where they form *en passant* synapses on the dendrites of pyramidal cells.

**Figure 1:**
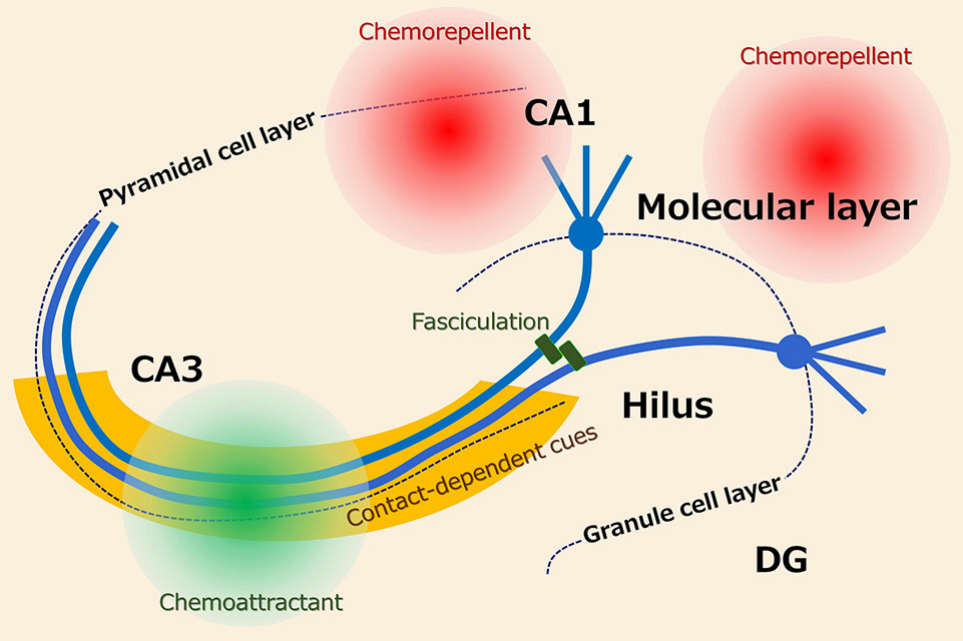
Possible axon guidance cues for hippocampal mossy fibers. It has been suggested that the projection of mossy fibers is ensured by several axon guidance cues including diffusible and contact-dependent cues.

The basically unidirectional projection of mossy fibers is ensured by several axon guidance cues (Figure 1). Studies that utilized entorhino-hippocampal slice cultures have revealed that the mossy fibers are attracted to CA3 by diffusible chemoattractants and repelled from CA1 by diffusible chemorepellents (2–4). Additionally, the activation of group II metabotropic glutamate receptors (5) and tropomyosin receptor kinase (Trk) receptors (6), phosphacan, which is probably derived from astrocytes (7), and polysialylated-neural cell adhesion molecule (PSA-NCAM), which is expressed on the mossy fibers (4), collaborate to keep the tightly bundled projections of the mossy fibers in the stratum lucidum. Furthermore, the classical axon guidance molecules ephrin (8), semaphorin (9, 10), and netrin (11) are also involved in the pruning and stratum lucidum-specific targeting of the mossy fibers.

Although several axon guidance cues act together to regulate the mossy fiber projections, under some epileptic conditions, the mossy fiber collaterals robustly branch out of the hilus and aberrantly, or reversely, project to the inner third of the molecular layer, in which the mossy fibers form excitatory synapses primarily on the granule cell dendrites (12–15). It has been reported that mossy fiber sprouting also develops in CA3 outside of the stratum lucidum. The misguidance of mossy fibers, including both robust axonal branching and reverse projection, i.e., mossy fiber sprouting, is a common pathological hallmark that is observed in individuals with mesial temporal lobe epilepsy (16, 17), and the proportion of granule cells that exhibit sprouted mossy fibers has been estimated to be approximately 60% (18). A number of experimental studies that mimic epilepsy *in vivo* and epileptiform seizures *in vitro* have replicated this mossy fiber sprouting (19); however, the axon misguidance mechanisms underlying mossy fiber sprouting have not been fully clarified. Clarifying the cellular and molecular mechanisms would lead to a future understanding of CNS network reorganization. Furthermore, in addition to mesial temporal lobe epilepsy, mossy fiber sprouting has been observed in diseases such as non-mesial temporal lobe epilepsy (20) and bipolar disorder (21). Thus, the study of mossy fiber sprouting may lead to the identification of common mechanisms in the pathological reorganization of neuronal circuits.

In this mini-review, we mainly focus on *in vitro* studies examining axon guidance mechanisms underlying mossy fiber sprouting in the molecular layer but not in CA3 (Table 1). Whether the dentate synapses provided by the sprouted mossy fibers are epileptogenic or conversely providing recurrent inhibition circuits is definitely an interesting issue (19, 22–24) but will not be mentioned here. A hypothesis on the involvement of inhibitory dentate basket neurons, i.e., the dormant basket cell hypothesis, can be found elsewhere (25, 26). Readers are also recommended to refer to a previous comprehensive review on mossy fiber sprouting (19) and the fundamental studies that describe the differences in mossy fiber reorganization between developing and adult rats (27, 28).

**Table 1:** Researches cited in the present review: possible roles of several molecules in axon guidance of mossy fibers.

**Role**	**Experimental model**	**Cellular and molecular factors**	**References**
**NORMAL PROJECTION**			
Chemoattraction to CA3	Slice culture	cAMP	2
	Slice culture	Not identified	(3, 4)
	Slice culture	Netrin and DCC	(11)
Chemorepulsion from CA1	Slice culture	cGMP	(2)
	Slice culture	Not identified	(3, 4)
Fasciculation (lamina-specific guidance)	Slice culture	PSA-NCAM	(4)
	Slice culture	mGluR2	(5)
	Slice culture	Trk receptors	(6)
	Slice culture	Phosphacan	(7)
	*In vivo* (transgenic mice)	Ephrin	(8)
	*In vivo* (transgenic mice)	Semaphorin	(9, 10)
**SPROUTING**			
Foundation	*In vivo* (kindling, rats)	Hilar cell loss	(29)
	*In vivo* (transgenic mice)	Mossy cell loss	(38)
Branching	Slice culture	BDNF	(48, 49)
	Cell culture	AMPK and mitochondrion	(56)
	Cell culture	cAMP	(55)
Reverse projection	Slice culture	Netrin-1 and UNC5	(11)
	Slice culture	RGMa	(65)
Lamina specific projection	*In vivo* (status epilepticus, rats)	Semaphorin	(71)
	Slice culture	Hyaluronan and CD44	(72)
Not identified	*In vivo* (BDNF injection, rat)	BDNF	(50)
	*In vivo* (transgenic mice)	BDNF	(51)
	*In vivo* (rapamycin injection, rats)	mTOR	(87, 88)

## Axon misguidance in mossy fiber sprouting

Regarding the cellular process of mossy fiber sprouting, we previously proposed the following 3-step axon guidance model (23): step 1, branching; step 2, reverse projection; and step 3, fasciculation. In addition to these steps, we will mention the foundation for the development of mossy fiber sprouting in the dentate gyrus and the contribution of adult-born granule cells to mossy fiber sprouting.

## The foundation

It has been proposed that mossy fiber sprouting is triggered by the loss of neurons in the hilus (29–33). The mossy cells, which are the most common hilar neurons, project their axons to the inner molecular layer to form excitatory synapses on granule cell dendrites (34, 35), which are also the primary target of sprouted mossy fibers. These axonal projection patterns imply that the sprouted mossy fibers replace the mossy cell projections in the inner molecular layer. This hypothesis is supported by the finding that the extent of mossy fiber sprouting correlates with the loss of mossy cells in a rat model of temporal lobe epilepsy and with the loss of hilar neurons in patients with mesial temporal lobe epilepsy (30, 36, 37). However, mossy cell loss alone is not sufficient to explain the induction of robust branching and the long-distance reverse projections to the molecular layer; we recognize mossy cell loss as the foundation for mossy fiber sprouting rather than as its trigger. The direct experimental evidence for this idea was reported in a study that used a conditional transgenic mouse line in which the diphtheria toxin receptor was selectively expressed in mossy cells (38). The authors of this study found that the mossy cells were extensively degenerated all along the longitudinal axis of the hippocampus 1 week after the injection of diphtheria toxin; however, neither spontaneous behavioral seizures nor mossy fiber sprouting was detected 5–6 weeks after the injection of the toxin. In contrast, an increase in GAD67 immunoreactivity began to appear 2 weeks after toxin injection, which indicated that axonal sprouting had occurred in the GABAergic interneurons. In addition, it has been reported in a rodent model of febrile seizures that mossy fiber sprouting occurs after hyperthermia-induced seizures without evident neuronal loss or aberrant neurogenesis (39). Therefore, it is likely that the loss of mossy cells alone is not sufficient and that additional seizure-related changes are necessary to induce mossy fiber sprouting. Thus, what triggers the branch formation?

## Branching

The mossy fibers first provide robust branches in the hilus to initiate the sprouting. Among the factors that could trigger axonal branching, the neurotrophin brain-derived neurotrophic factor (BDNF) has received the most attention (40). The expression of BDNF mRNA (41) and protein (42), as well as its high-affinity receptor TrkB (43), are upregulated in hippocampal tissues from human epileptic brains. In rodents, the highest level of BDNF occurs in the mossy fiber pathway, where it is localized in the mossy fiber terminals (44), and the level of BDNF in these terminals is highly upregulated after seizure onset (45). Because BDNF is a potential morphoregulator of axonal branching (46, 47), it is possible that seizure activity induces the release of BDNF from mossy fiber terminals, thereby resulting in branch formation in nearby mossy fiber shafts. Koyama et al. tested this hypothesis using organotypic slice cultures and dispersed cultures of dentate granule cells (48). These authors cultured entorhino-hippocampal slices with the GABA_A_ receptor antagonist picrotoxin to induce neuronal hyperactivity. Robust mossy fiber sprouting was induced when the slice cultures were treated with picrotoxin for 10 days. In the picrotoxin-treated slice cultures, BDNF protein was upregulated specifically in the mossy fiber pathway. Mossy fiber sprouting was prevented by the application of the Trk blocker K252a or function-blocking anti-BDNF antibody. These authors further found that a BDNF-containing bead placed on the hilus induced sprouting and concluded that BDNF induces the branching out of hilar axonal shafts. Additionally, the BDNF-induced axonal branching was blocked when truncated TrkB receptors that lack the intracellular tyrosine kinase domain were expressed in cultured granule cells. These results indicate that BDNF could trigger mossy fiber sprouting. Finally, increased expression of BDNF induced the branching of mossy fibers in cultured slices (49), and intrahippocampal infusion of BDNF induced mossy fiber sprouting in rats (50). Additionally, electroconvulsive seizure-induced mossy fiber sprouting was attenuated in BDNF heterozygote knockout mice (51). Taken together, these results indicate that BDNF likely plays the role of a trigger of the induction of the first step of mossy fiber sprouting, that is, the initial branching of the mossy fiber collaterals in the hilus. However, it should be noted that several studies have questioned the role of BDNF in mossy fiber sprouting. For example, mossy fiber sprouting was induced in slice cultures prepared from BDNF knockout mice (52), and neither BDNF overexpression (53) nor BDNF infusion (51) resulted in mossy fiber sprouting in mice.

The BDNF-induced signaling pathway that is involved in mossy fiber sprouting has not been fully identified. The axonal branching induced by neurotrophins, including BDNF, is partly mediated by 3′,5′-cyclic adenosine monophosphate (cAMP) (54). An optogenetic method was recently employed to reveal that an increase in the intracellular cAMP levels of granule cells alone can induce the branching and elongation of axons. These authors of this study photo-activated cultured granule cells that expressed photo-activated adenylyl cyclase (PAC), which produces cAMP in response to exposure to blue light (55). Interestingly, AMP, which is the product of the degradation of cAMP that is catalyzed by the enzyme phosphodiesterase, activates AMP-activated protein kinase (AMPK) in a neuronal activity-dependent manner and enhances the anterograde transport of mitochondria into the axons of cultured granule cells (56). Real-time imaging of axonal morphology and mitochondrial distribution has revealed that mitochondrial localization in axons precedes axonal branching. Thus, it is possible that BDNF-induced mossy fiber branching is mediated by the cAMP-dependent localization of axonal mitochondria.

It should be noted that there are several molecules that are not reviewed here but have been investigated as candidates to induce outgrowth and branching of mossy fibers. For example, 9-*O*-acetylated gangliosides, whose expression has been shown to correlate with axonal growth in several brain regions, did not contribute to mossy fiber sprouting in adult epileptic rats (57).

## Reverse projection

In contrast to the foundation and branching in mossy fiber sprouting, the cellular and molecular processes that mediate the reverse projection to the molecular layer have not yet been clarified. Muramatsu and colleagues examined the possible involvement of the axon guidance molecule netrin-1 in reverse projection (11) because it was shown that netrin-1 expression was increased in the dentate gyrus after kainic acid-induced seizures in the rat (58). The netrins serve as secretory proteins that regulate axon guidance (59) and serve as ligands for the transmembrane immunoglobulin superfamily proteins deleted in colorectal cancer (DCC) and uncoordinated-5 (UNC5) (60). The combination patterns of DCC and UNC5 on the growth cone surface of growing axons determine whether netrins act as attractants or repellents: the presence of DCC attracts the axons to netrins (61, 62), whereas the presence of both DCC and UNC5 repels axons from netrins (63). UNC5 alone also mediates netrin-induced axonal repulsion (64).

Inspired by the dual guidance property of netrin-1, Muramatsu and colleagues examined the role of netrin-1 and its receptors in the reverse projection of mossy fibers during the establishment of mossy fiber sprouting (11). Netrin-1 antibody or DCC knockdown attenuated mossy fiber growth toward CA3 in a slice overlay assay in which immature granule cells are dispersed and cultured on organotypic entorhino-hippocampal slices. Further, drug-induced neuronal hyperactivity did not affect the expression levels of DCC on the mossy fiber growth cone surface, but it increased the expression levels of UNC5A in a cAMP-dependent manner. Finally, UNC5A knockdown in granule cells blocked activity-dependent mossy fiber sprouting in slice cultures. Thus, it was suggested that netrin-1 attracts mossy fibers to CA3 via DCC, while netrin-1 repels mossy fibers via UNC5A under hyperexcitable conditions.

Additionally, repulsive guidance molecule a (RGMa), whose expression was high in the granule cell layer, blocked activity-dependent mossy fiber sprouting in hippocampal slice cultures by preventing the sprouted collaterals from invading the inner molecular layer (65). Shetty and colleagues successfully suppressed mossy fiber sprouting *in vivo* by transplanting CA3 cell grafts into the hippocampus (66–68) in a manner that was possibly mediated by competition between the diffusible chemoattractants derived from the CA3 cell grafts and axon guidance cues that mediate the reverse projection.

After the reverse-traveling mossy fibers enter the molecular layer, they are tightly confined in the inner molecular layer and usually do not invade the middle or outer molecular layers [but see the study from Babb and colleagues (30), which showed possible synaptic reorganization in the middle molecular layer]. This mossy fiber trajectory suggests that both attractive and repulsive axon guidance molecules regulate the laminar specificity of the mossy fiber growth in the molecular layer. One such molecule would be Sema3A, which serves as a chemorepellent via a receptor complex composed of neuropilin-1 and plexinA (69, 70). (71) reported that the sprouting of mossy fibers is correlated with the downregulation of Sema3A mRNA that is observed in the stellate cells of the entorhinal cortex layer II in epileptic rats. Thus, it was hypothesized that stellate cells project their axons to the molecular layer and provide a chemorepulsive gradient of Sema3A in the molecular layer, preventing mossy fiber collaterals from invading the molecular layer. It has also been suggested that the extracellular matrix component hyaluronan and its primary receptor CD44 contribute to the development of kainic acid-induced mossy fiber sprouting in the inner molecular layer of cultured hippocampal slices (72). However, the mechanisms that regulate the specific axonal targeting to the inner molecular layer remain to be clarified.

## Fasciculation and neurogenesis

When the pioneer mossy fibers complete their extension to the molecular layer, it is possible that the following mossy fibers fasciculate with them in a manner guided by contact-dependent axon guidance cues, such as PSA-NCAM, which is expressed on mossy fibers. Based on an organotypic coculture system, it has been suggested that PSA-NCAM serves as a contact cue for the following mossy fibers when diffusible chemoattractants from CA3 are no longer available in the late developmental stage (4).

Adult neurogenesis in the dentate gyrus could also account for additional fasciculation to the sprouted mossy fibers, as it has been suggested that adult-born granule cells are the main source of sprouted mossy fibers after status epilepticus (SE) (73). Dentate granule cells are continuously generated throughout life, and adult-born granule cells comprise approximately 6% of the total granule cell population each month in adult rats (74); thus, a portion of the mossy fibers may undergo continuous turnover over a period of weeks (75). It has been suggested that both neonatal- and adult-born granule cells contribute to the sprouted mossy fibers with synaptic boutons in the inner molecular layer after pilocarpine-induced SE in adult rats (76). A pioneering work reported that the elimination of adult-born granule cells by whole-brain X-irradiation performed 1 day before and 4 days after SE did not block mossy fiber sprouting 4 weeks later in the rat, suggesting that granule cells born after SE do not provide mossy fiber sprouting (77). Moreover, retrovirus-mediated cell labeling studies have successfully identified the morphological features of adult-born granule cells before and after SE and have thus advanced our understanding of the role of neurogenesis in mossy fiber sprouting. The granule cells born up to 4 weeks before SE (73, 78) or up to 4 days after SE exhibited sprouted mossy fibers, whereas the mature granule cells born ≥ 7 weeks before SE did not (73). Interestingly, adult-born granule cells require more than 4 and fewer than 10 weeks to produce mossy fiber sprouting (73). After SE, cell proliferation in the dentate gyrus increases by 5 to 10-fold and persists for several weeks. Moreover, in rodents, 75–90% of these cells become mature granule cells within 4 weeks (78–80). Thus, although it remains unclear whether all adult-born granule cells exhibit sprouted mossy fibers, adult neurogenesis seems to reinforce mossy fiber sprouting for at least several weeks. It has been reported that the ablation of adult-born granule cells for 4–5 weeks until the time of SE induction did not decrease the SE-induced sprouting at 7–12 weeks post-SE (81, 82). Thus, it is likely that adult-born granule cells sprout maximally during a relatively limited and specific post-SE period. It is also possible that axons from mature granule cells exhibit robust sprouting, compensating for the loss of sprouting from ablated adult-born granule cells, as it has also been shown that mature granule cells can also contribute to sprouting (76). In addition, we need to be careful to translate these findings into the human brain because the differences in the systems of neurogenesis between rodents and humans need to be further studied (83, 84).

## Conclusion

**Figure 2:**
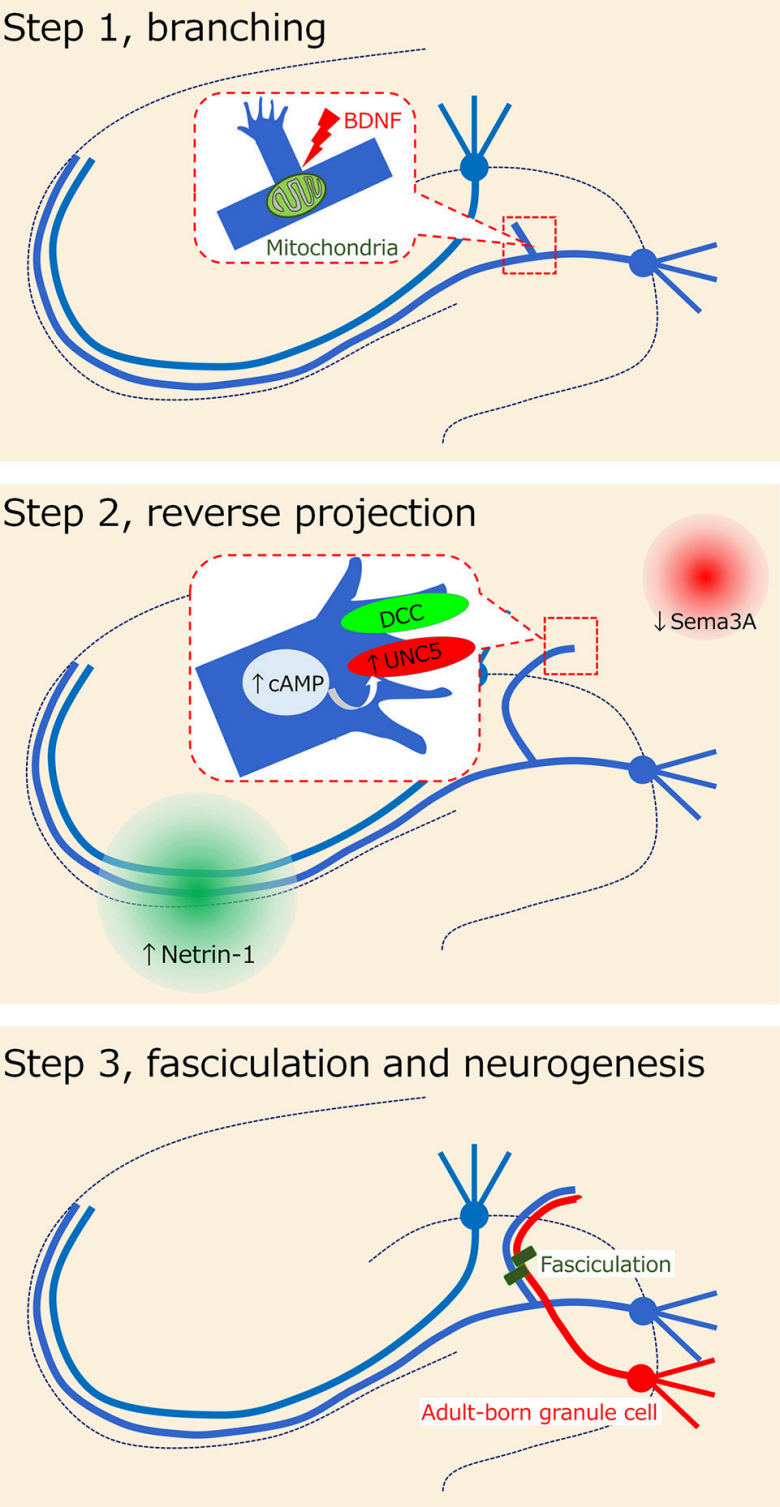
Hypothesis on axon misguidance in mossy fiber sprouting. It has been previously proposed that the cellular process of mossy fiber sprouting consists of the 3-step axon guidance model (23): step 1, branching; step 2, reverse projection; and step 3, fasciculation. Several possible molecules such as BDNF and netrin receptors have been suggested to be involved in each step.

The misguidance of mossy fibers to the inner molecular layer has been demonstrated to be regulated by several molecules (Figure 2). It should be noted, however, that the axon guidance molecules described in the present review may also affect other axons or synapses and then induce mossy fiber sprouting in response. Intriguingly, the mossy fiber collaterals are tightly guided to and confined to the inner molecular layer, even though this is not the original target in non-epileptic conditions. Thus, the “mis”-guidance of mossy fibers might be a necessary process for the homeostasis of dentate neural circuits under epileptic conditions. The idea also depends on whether mossy fiber sprouting is reversible. A possible pharmacological modulator of mossy fiber sprouting could be cycloheximide, a protein synthesis inhibitor, because it was shown to inhibit seizure-induced mossy fiber sprouting (85). However, it should be noted that another study failed to reproduce the blocking effect of cycloheximide on mossy fiber sprouting (86). Mammalian target of rapamycin (mTOR) signaling may also be a candidate because its inhibition by rapamycin prevented mossy fiber sprouting and reduced seizures in a rodent model of acquired epilepsy (87, 88). Experiments that conditionally and precisely modulate the occurrence of mossy fiber sprouting by regulating the mossy fiber axon guidance system are necessary to determine whether mossy fiber sprouting is merely epileptogenic or eventually homeostatic.

## Author contributions

RK and YI discussed and wrote the manuscript.

### Conflict of interest statement

The authors declare that the research was conducted in the absence of any commercial or financial relationships that could be construed as a potential conflict of interest.
